# Supplementary material for: The late positive component in recognition memory is linked to mnemonic evidence accumulation
Source: Imaging Neurosci (Camb). 2026 Oct 13;4:IMAG.a.1389. doi: 10.1162/IMAG.a.1389 (PMC13662653; doi:10.1162/IMAG.a.1389)
Supplement: Supplementary file 1 [file IMAG.a.1389_supp.pdf]

Supplementary Material for: The Late Positive Component in Recognition Memory is Linked to Mnemonic Evidence Accumulation

Jie Sun, Daniel Feuerriegel, Mia Nightingale, Adam F. Osth

Melbourne School of Psychological Sciences, The University of Melbourne

Corresponding Author:

Jie Sun

Jies3@student.unimelb.edu.au

Melbourne School of Psychological Sciences, Redmond Barry Building, The University of Melbourne, 3010, Australia.

Supplementary Material

Supplementary Figure 1. Recovery of best fitting parameters and variance-explained measures from the model that links LPC amplitude with drift rate.

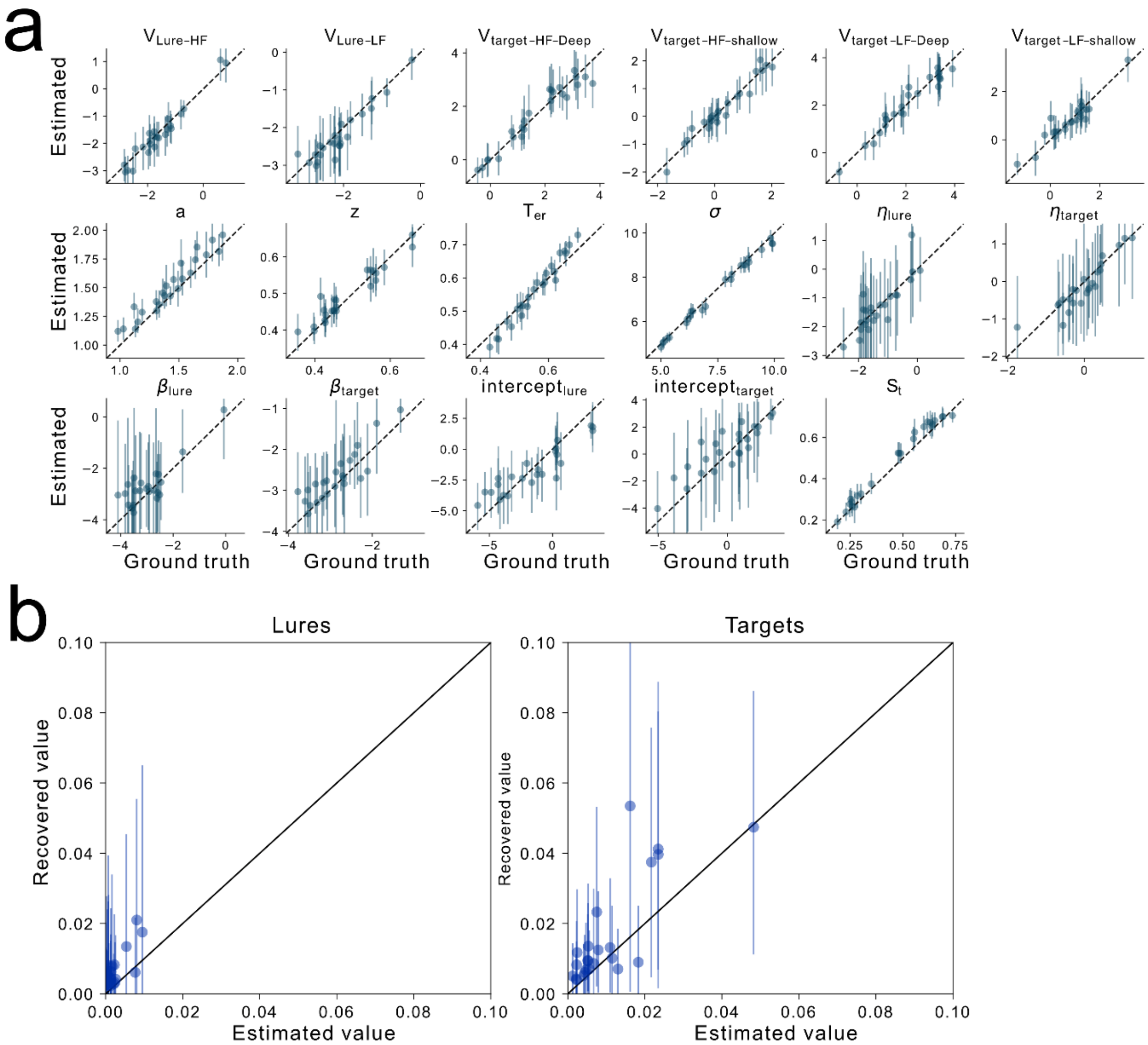

Note. a. Parameter recovery of the best fitting parameters for each participant. The best fitting parameters were the most frequent in the posterior distributions, which were plotted against the mean of the recovered parameters across 500 samples. The error band represents the 95% highest density interval for the recovered means for parameters across the 500 samples. b. Recovery of the variance-explained measured across 500 samples.

Supplementary Figure 2. Recovery of best fitting parameters and variance-explained measures from the model that links LPC amplitudes in three time windows with drift rate.

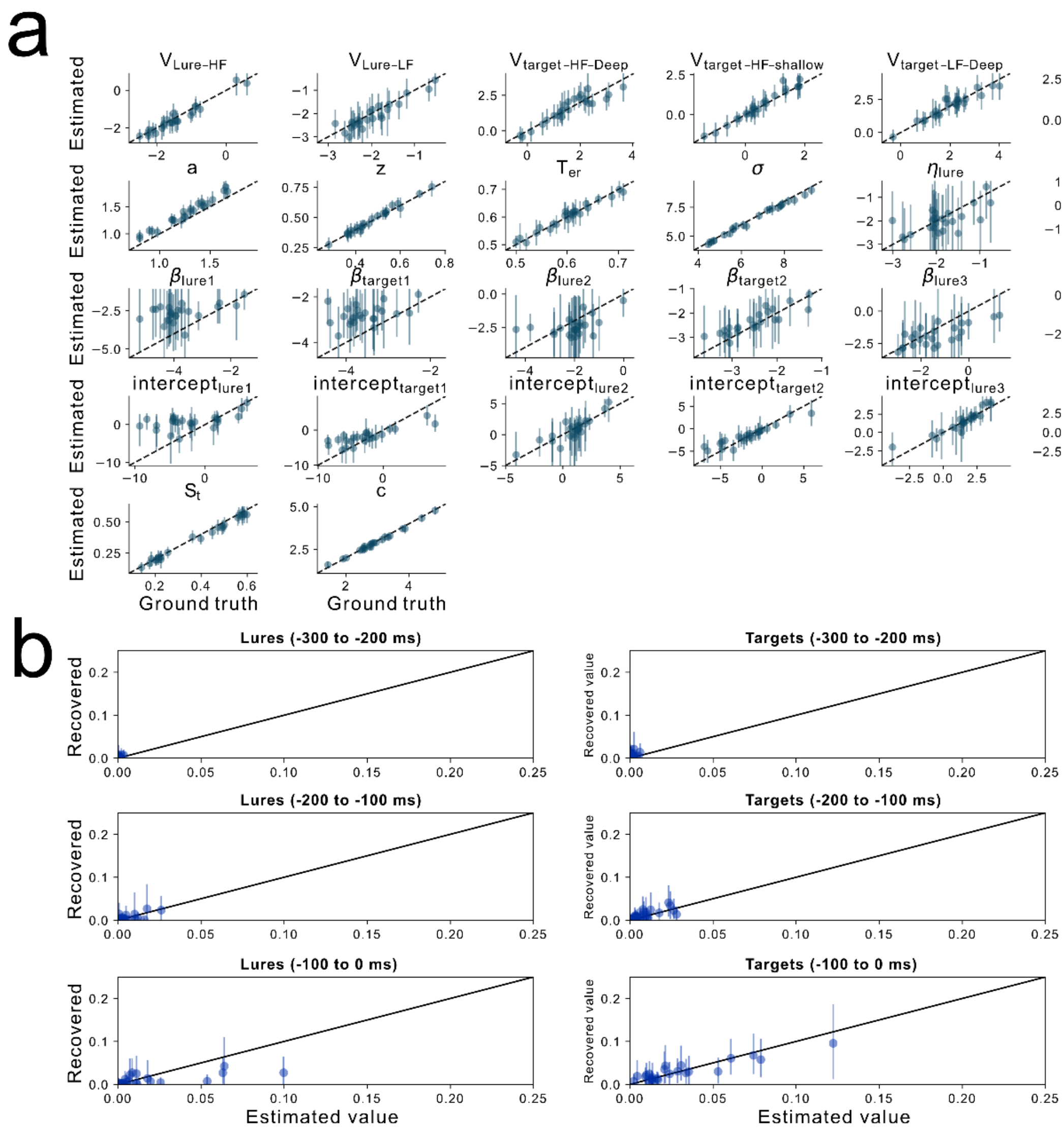

Note. a. Parameter recovery of the best fitting parameters for each participant. The best fitting parameter were the most frequent in the posterior distributions, which were plotted against the mean of the recovered parameters across 500 samples. The error band represents the 95% highest density interval for the recovered means for parameters across the 500 samples. b. Recovery of the variance-explained measured across 500 samples for each time window.

Supplementary Figure 3. Recovery of best fitting parameters and variance-explained measures from the model that links drift rate with LPC and visual P1 amplitudes.

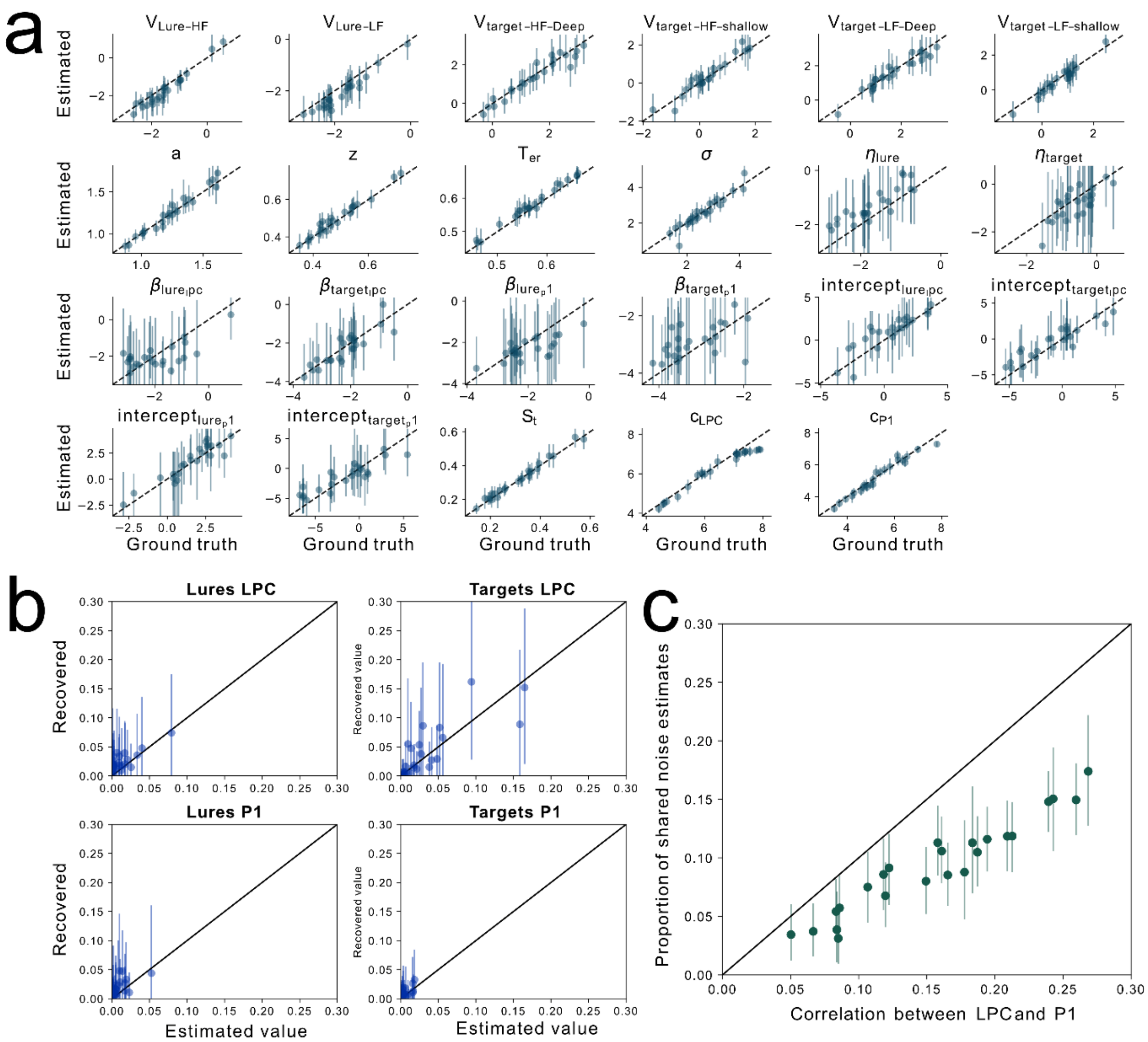

Note. a. Parameter recovery of the best fitting parameters for each participant. The best fitting parameter were the most frequent in the posterior distributions, which were plotted against the mean of the recovered parameters across 500 samples. The error band represents the 95% highest density interval for the recovered means for parameters across the 500 samples. b. Recovery of the variance-explained measured across 500 samples for LPC and P1visual P1 amplitudes. c. The correlation between the model estimated shared noise between LPC and P1 and the trial-by-trial correlation between LPC and P1 across participants.

Supplementary Figure 4. Recovery of best fitting parameters and variance-explained measures from the model that links non-decision time with LPC amplitudes.

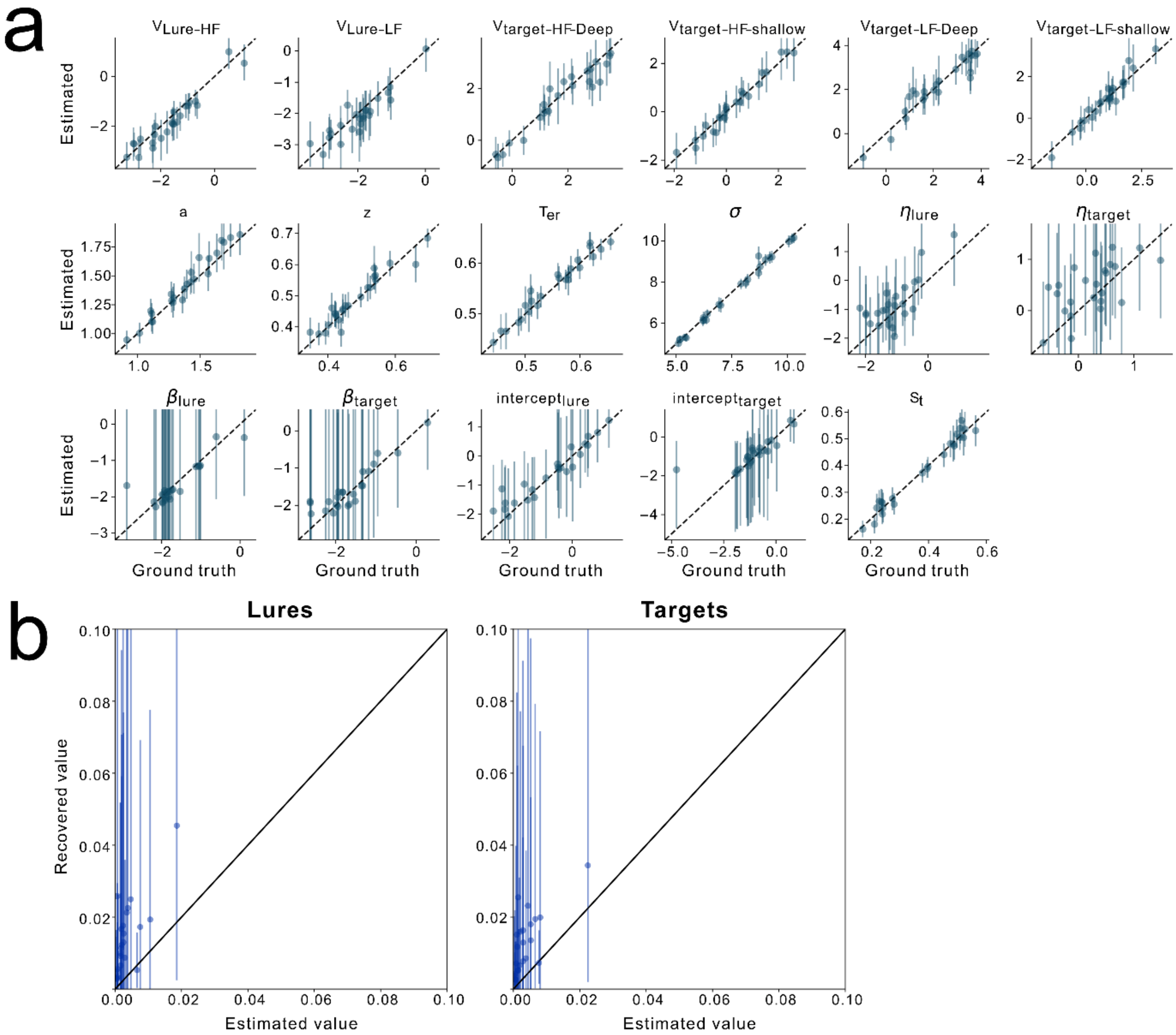

Note. a. Parameter recovery of the best fitting parameters for each participant. The best fitting parameter were the most frequent in the posterior distributions, which were plotted against the mean of the recovered parameters across 500 samples. The error band represents the 95% highest density interval for the recovered means for parameters across the 500 samples. b. Recovery of the variance-explained measured across 500 samples.

Supplementary Figure 5. Model predictions for the model linking drift rate with three time windows of LPC amplitudes.

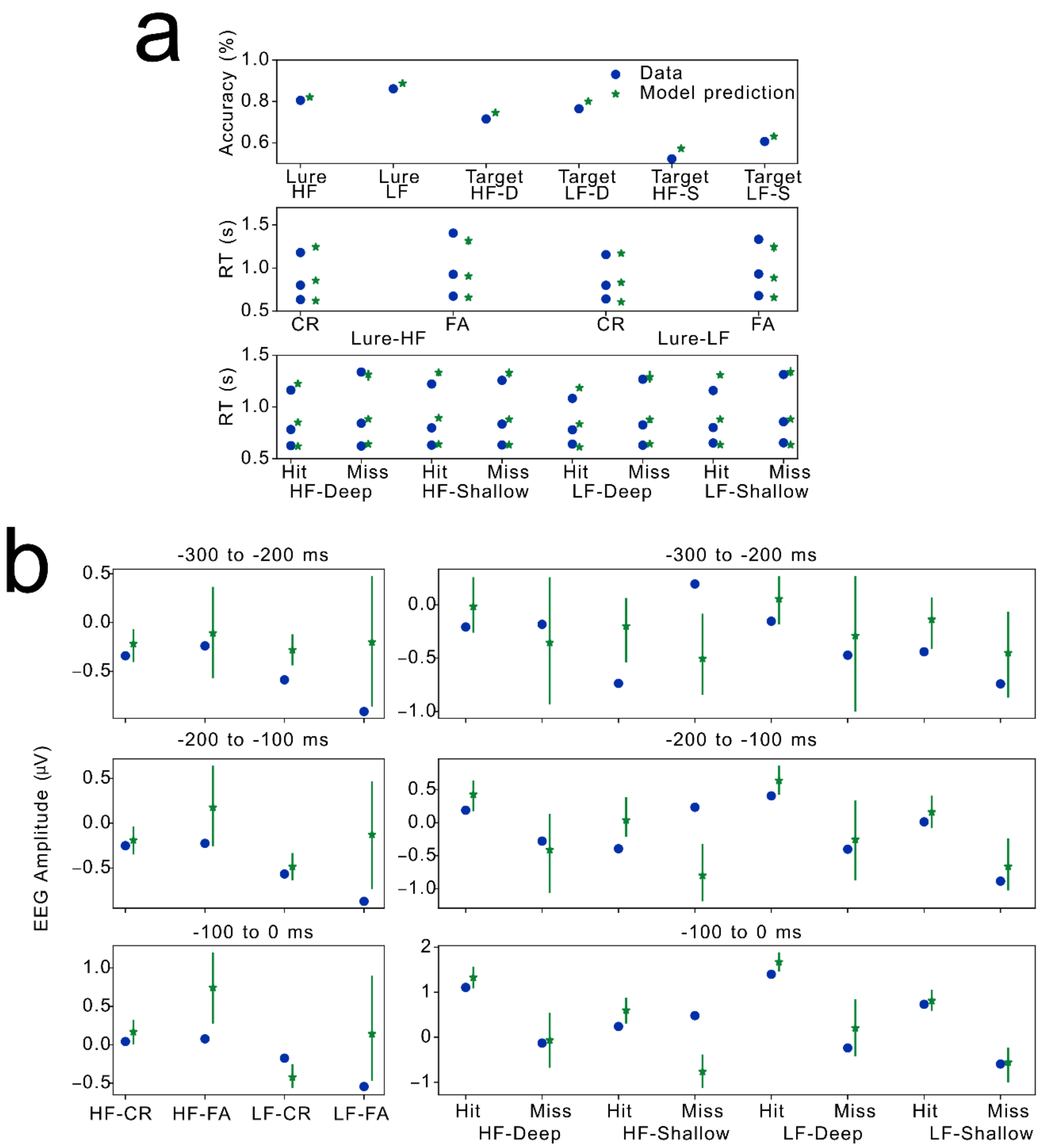

Note. a. Model predictions of behavioural data based on the best fitting parameters across participants. b. Model predictions of mean LPC amplitude across three time windows data based on the best fitting parameters across participants. The error band represents the 95% highest density interval across 200 samples generated from the best fitting parameters.

Supplementary Figure 6. Model predictions for the model linking drift rate with LPC and visual P1 amplitudes.

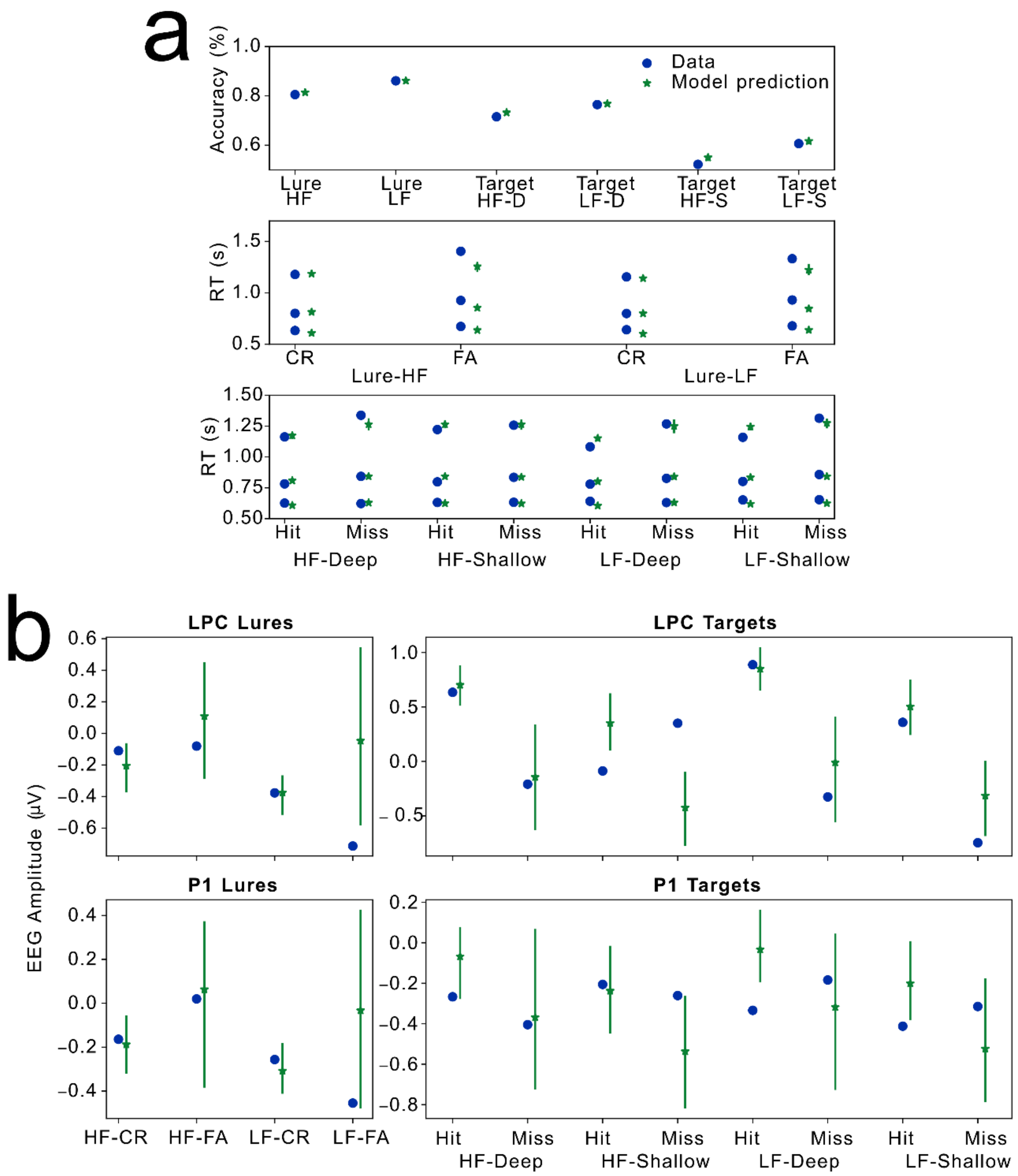

Note. a. Model predictions of behavioural data based on the best fitting parameters across participants. b. Model predictions of mean LPC and P1visual P1 amplitudes based on the best fitting parameters across participants. The error band represents the 95% highest density interval across 200 samples generated from the best fitting parameters.

Supplementary Figure 7. Model predictions for the model linking non-decision time with LPC amplitudes.

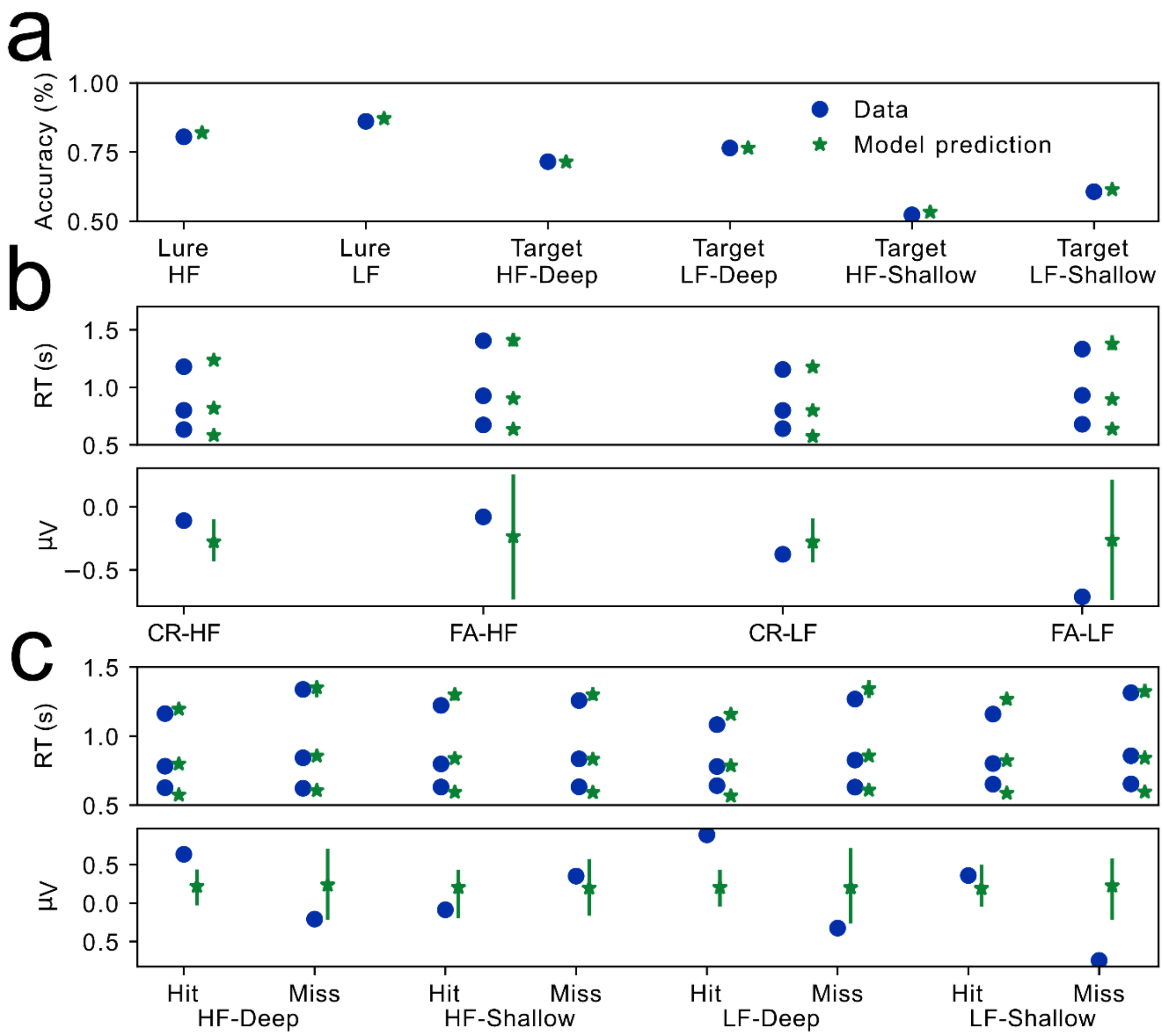

Note. a. Model predictions of accuracy based on the best fitting parameters across participants. b. Model predictions of RTs and mean LPC amplitudes for lures. c. Model predictions of RTs and mean LPC amplitudes for targets. The error band represents the 95% highest density interval across 200 samples generated from the best fitting parameters.

Supplementary Figure 8. Comparisons of parameter estimates in Model 2 from models either trained on small or large number of trials per sample.

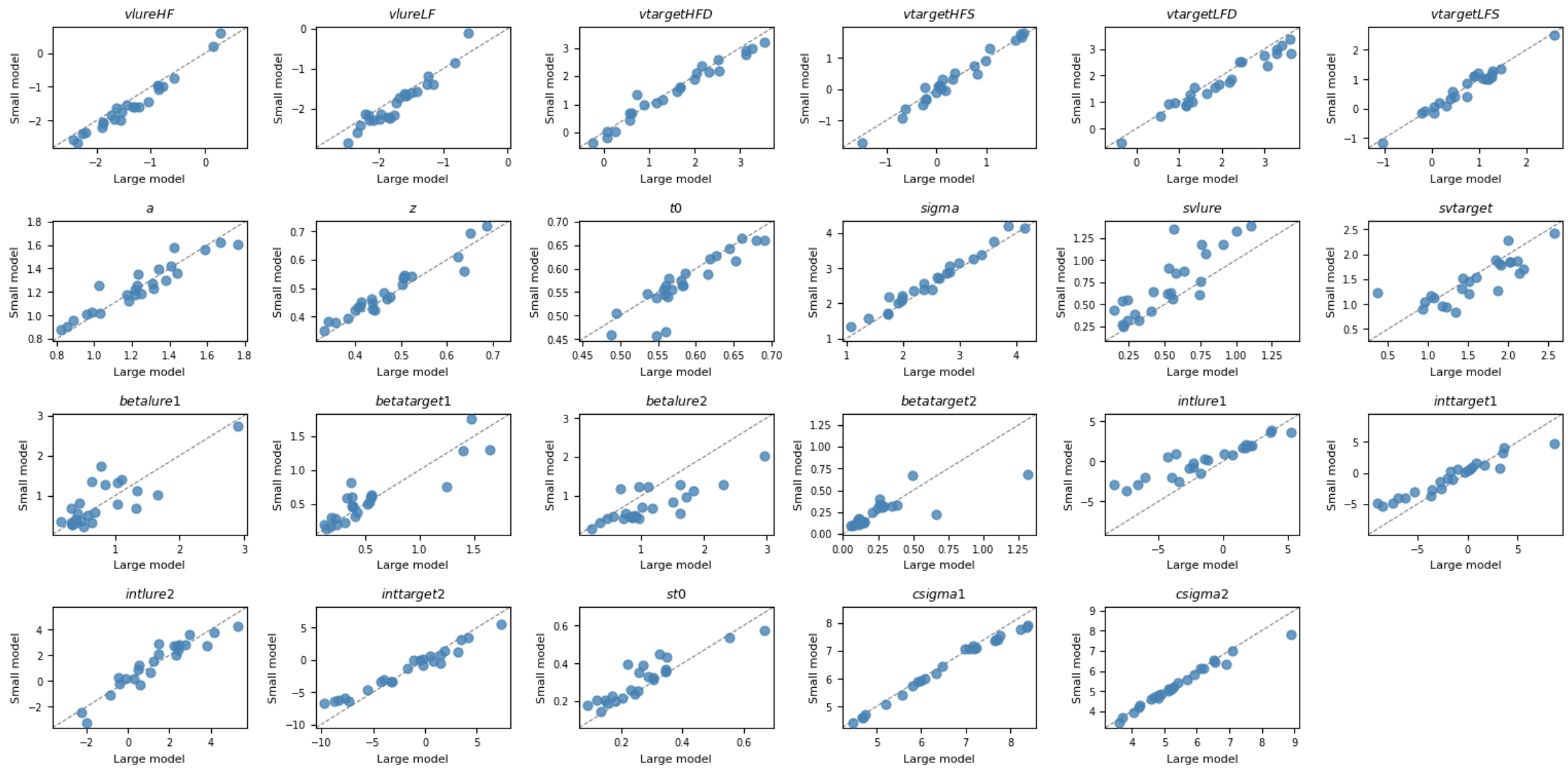

Note. The parameters were based on the median values from the posterior distributions after model fit to the experimental data. The small-sample model was trained on 300-500 trials per simulation; the large-sample model was trained on 1800-2400 trials per simulation.

Supplementary Figure 9. Examples of parameter distributions drawn from either a uniform distribution (left), a uniform distribution after transformation using a sigmoid function (middle), or a normal distribution after transformation using a sigmoid function (right).

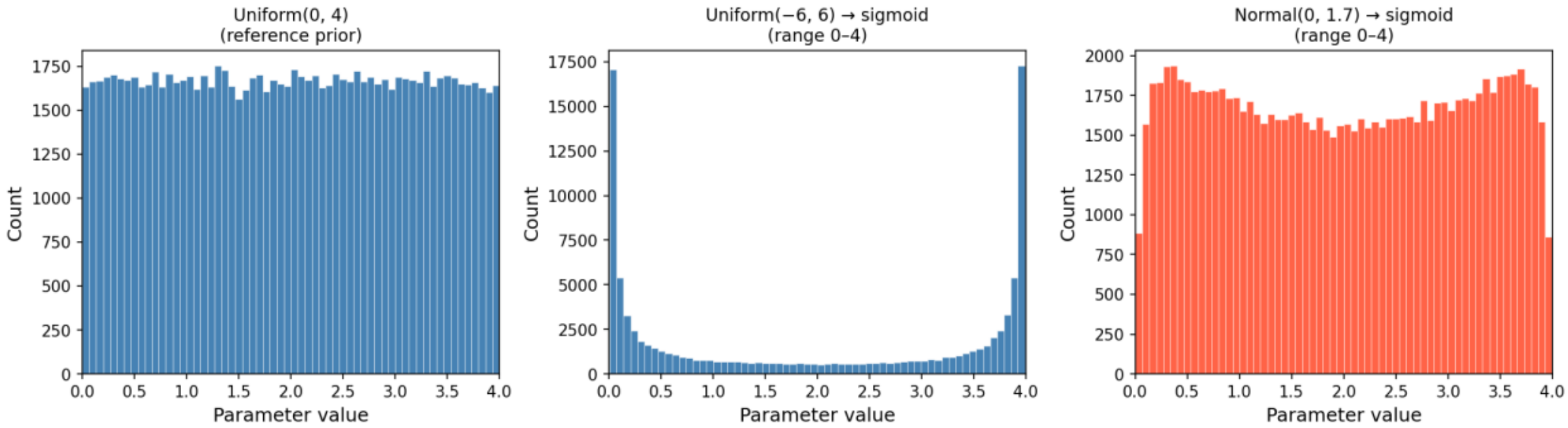

Supplementary Figure 10. Variance-explained measures for the model that jointly predicts the LPC amplitude and also the pre-response amplitude at channel F6 after regressing out the LPC amplitude.

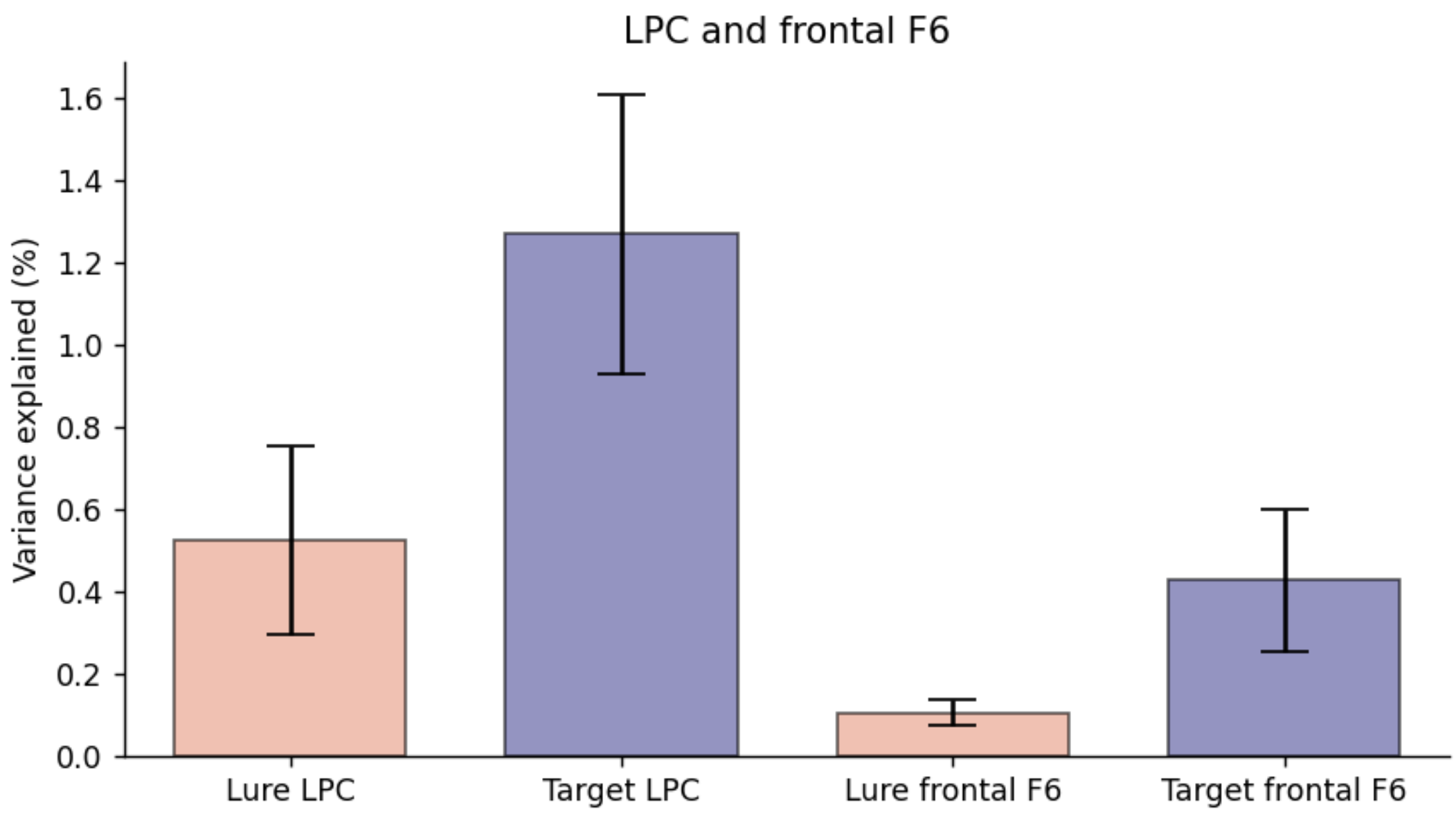

Note. The error bar represents the 95% HDI.

Supplementary Figure 11. With the data after subtracting the stimulus-locked components, variance-explained measures for the model simultaneously predicts pre-response LPC amplitudes and LPC slopes estimated from -300 to 0 ms around the response.

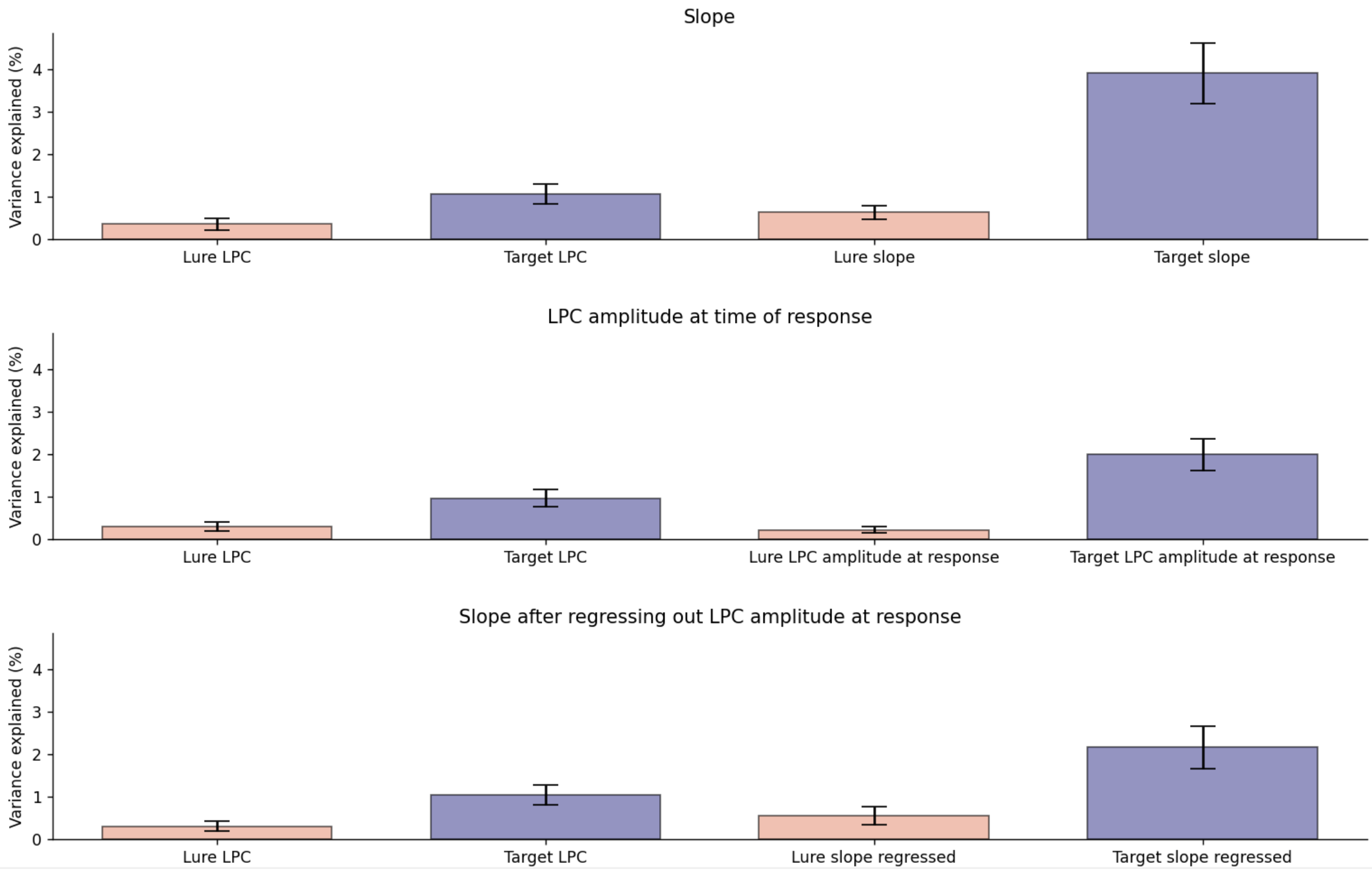

Note. The model was set up the same way as the Model 2 but replacing prediction of P1 with the LPC slopes. The slope measures were estimated from -300 to 0 ms relative to the response and were rescaled to be consistent the range of predicted values of the neural component from initial training. The top panel shows the variance-explained measures across targets and lures for the prediction of the LPC amplitude and slope, separately. The middle panel shows the variance-explained measures across targets and lures for the prediction of the averaged pre-response LPC amplitude and the LPC amplitude at the time of the response, separately. The bottom panel shows the variance-explained measures across targets and lures for the prediction of the averaged pre-response LPC amplitude and residual of slope measures after regressing out the LPC amplitude at the time of response.

Supplementary Table 1. Parameters included in each model

Models	Parameters
Model 1	$V_{HF\text{-}L}$, $V_{LF\text{-}L}$, $V_{HF\text{-}D\text{-}T}$, $V_{LF\text{-}D\text{-}T}$, $V_{HF\text{-}S\text{-}T}$, $V_{LF\text{-}S\text{-}T}$, a, z, T_{er}, σ, η_L, η_T, β_L, β_T, β_{oL}, β_{oT}, S_t
Model 2	$V_{HF\text{-}L}$, $V_{LF\text{-}L}$, $V_{HF\text{-}D\text{-}T}$, $V_{LF\text{-}D\text{-}T}$, $V_{HF\text{-}S\text{-}T}$, $V_{LF\text{-}S\text{-}T}$, a, z, T_{er}, σ, η_L, η_T, $\beta_{t1\text{-}L}$, $\beta_{t2\text{-}L}$, $\beta_{t1\text{-}L}$, $\beta_{t1\text{-}T}$, $\beta_{t2\text{-}T}$, $\beta_{t3\text{-}T}$, $\beta_{o\text{-}t1\text{-}L}$, $\beta_{o\text{-}t2\text{-}L}$, $\beta_{o\text{-}t3\text{-}L}$, $\beta_{o\text{-}t1\text{-}T}$, $\beta_{o\text{-}t2\text{-}T}$, $\beta_{o\text{-}t3\text{-}T}$, S_t, c
Model 3	$V_{HF\text{-}L}$, $V_{LF\text{-}L}$, $V_{HF\text{-}D\text{-}T}$, $V_{LF\text{-}D\text{-}T}$, $V_{HF\text{-}S\text{-}T}$, $V_{LF\text{-}S\text{-}T}$, a, z, T_{er}, σ, η_L, η_T, $\beta_{LPC\text{-}L}$, $\beta_{P1\text{-}L}$, $\beta_{LPC\text{-}T}$, $\beta_{P1\text{-}T}$, $\beta_{o\text{-}t1\text{-}L}$, $\beta_{o\text{-}LPC\text{-}L}$, $\beta_{o\text{-}P1\text{-}L}$, $\beta_{o\text{-}LPC\text{-}T}$, $\beta_{o\text{-}P1\text{-}T}$, S_t, c_{LPC}, c_{P1}
Model 4	$V_{HF\text{-}L}$, $V_{LF\text{-}L}$, $V_{HF\text{-}D\text{-}T}$, $V_{LF\text{-}D\text{-}T}$, $V_{HF\text{-}S\text{-}T}$, $V_{LF\text{-}S\text{-}T}$, a, z, T_{er}, σ, η_L, η_T, β_L, β_T, β_{oL}, β_{oT}, S_t

Note. *Subscript HF = high word frequency, LF = low word frequency, S = Shallow encoding, D = Deep encoding, T = target, L = lure. Subscripts t1 – t3 refer to the three100-ms time windows from -300 to 0 ms around the response.*

Supplementary Table 2. Model 1 median parameter estimates of the posterior distributions across participants.

Participant Number	$V_{\text{HF-L}}$	$V_{\text{LF-L}}$	$V_{\text{HF-D-T}}$	$V_{\text{LF-D-T}}$	$V_{HF\text{-}S\text{-}T}$	$V_{\text{LF-S-T}}$	a	z	T_{er},	σ	η_{L}	η_{T}	β_{L}	β_{T}	β_{oL}	β_{oT}	S_{t}
1	-2.16	-2.61	1.23	1.45	1.83	0.87	1.15	0.58	0.54	5.00	-0.73	0.28	-3.50	-3.60	-1.13	0.89	0.48
2	-1.16	-2.11	2.53	2.02	3.37	3.13	1.53	0.55	0.61	9.83	-0.92	-0.31	-3.43	-2.68	0.36	-0.36	0.26
3	-2.79	-2.68	2.79	-0.19	3.34	1.56	1.13	0.54	0.59	6.16	-1.48	0.10	-3.22	-3.09	-5.92	-0.83	0.19
4	-1.26	-2.10	2.29	1.60	3.31	1.25	1.87	0.45	0.56	8.66	0.09	0.43	-3.70	-3.53	0.67	2.17	0.25
5	-1.93	-2.23	0.31	-0.12	0.68	0.21	1.49	0.56	0.65	8.52	-0.22	0.90	-2.58	-2.89	0.40	0.82	0.60
6	-2.71	-2.74	3.46	0.88	3.91	1.41	1.43	0.66	0.69	5.12	-1.97	0.12	-3.72	-2.75	-2.03	1.05	0.23
7	-2.84	-2.89	2.64	-0.96	2.48	-0.24	1.60	0.40	0.48	5.37	-1.65	-0.09	-3.82	-3.35	-2.39	-1.89	0.35
8	-1.76	-2.53	1.16	0.79	0.93	1.10	1.64	0.55	0.54	6.40	-0.68	0.34	-3.55	-3.77	-1.21	-1.53	0.64
9	-1.76	-1.82	-0.28	-1.07	-0.74	-1.34	1.84	0.43	0.51	9.91	-1.76	-0.56	-4.09	-2.86	3.19	0.86	0.69
10	-1.91	-1.90	-0.10	0.06	1.51	1.45	0.98	0.35	0.58	5.29	-1.76	-0.02	-2.74	-3.53	-3.87	0.87	0.49
11	-0.71	-0.88	0.84	0.46	1.18	0.59	1.65	0.45	0.52	8.11	-1.72	-0.67	-1.64	-2.35	3.18	3.16	0.69
12	-1.75	-1.51	2.17	0.11	3.42	1.05	1.36	0.42	0.52	7.84	-1.91	-0.39	-2.65	-2.28	0.24	2.24	0.62
13	-1.30	-2.34	-0.08	-0.80	0.33	-0.60	1.78	0.46	0.45	9.43	-1.25	0.46	-2.94	-3.48	-4.29	-3.86	0.63
14	-0.80	-1.26	2.23	1.23	2.12	0.81	1.19	0.44	0.62	8.71	-1.10	1.24	-3.26	-3.32	0.30	1.46	0.55
15	-1.66	-2.24	1.15	-0.37	1.80	0.18	1.45	0.43	0.49	5.08	-1.58	0.06	-3.51	-3.07	-3.54	-0.58	0.64
16	-1.96	-2.60	3.12	-0.08	2.10	0.14	1.31	0.46	0.66	9.89	-1.91	1.05	-3.48	-3.16	-0.82	1.96	0.30

17	-2.52	-2.10	-0.46	-0.21	1.15	1.21	1.38	0.40	0.65	8.08	-1.38	-0.30	-3.02	-2.11	-1.64	-2.86	0.26
18	-1.92	-2.06	2.21	-0.14	2.98	0.60	1.31	0.46	0.63	8.87	-2.51	-0.41	-0.08	-1.33	3.05	3.28	0.25
19	-1.37	-2.21	3.19	0.09	3.38	1.25	1.03	0.41	0.43	6.12	-1.57	-0.22	-2.51	-3.20	-4.30	0.34	0.48
20	-1.18	-2.08	0.78	0.13	1.18	0.32	1.12	0.56	0.62	6.29	-1.84	-1.76	-2.61	-2.71	-4.16	-0.94	0.74
21	0.80	-0.23	1.40	1.67	1.90	1.24	1.39	0.55	0.45	8.85	-0.16	-0.56	-2.55	-1.90	-4.85	-5.04	0.65
22	-2.44	-3.18	3.07	-1.68	3.36	0.02	1.73	0.43	0.59	6.83	-1.00	0.40	-2.91	-2.53	0.29	1.54	0.27
23	0.61	-1.28	1.24	1.80	1.15	1.27	1.52	0.66	0.52	6.32	-0.19	-0.69	-3.55	-2.67	-3.22	-2.91	0.56
24	-1.60	-2.71	3.74	0.39	3.33	1.22	1.33	0.45	0.57	6.96	-1.83	0.20	-2.46	-2.42	-5.35	-1.88	0.28

Supplementary Table 3. Model 2 median parameter estimates of the posterior distributions across participants.

p	$V_{HF\text{-}L}$	$V_{LF\text{-}L}$	$V_{HF\text{-}D\text{-}T}$	$V_{LF\text{-}D\text{-}T}$	$V_{HF\text{-}S\text{-}T}$	$V_{LF\text{-}S\text{-}T}$	a	z	T_{er},	σ	η_L	η_T	$\beta_{t1\text{-}L}$	$\beta_{t2\text{-}L}$	$\beta_{t1\text{-}L}$	$\beta_{t1\text{-}T}$	$\beta_{t2\text{-}T}$	$\beta_{t3\text{-}T}$	$\beta_{o\text{-}t1\text{-}L}$	$\beta_{o\text{-}t2\text{-}L}$	$\beta_{o\text{-}t3\text{-}L}$	$\beta_{o\text{-}t1\text{-}T}$	$\beta_{o\text{-}t2\text{-}T}$	$\beta_{o\text{-}t3\text{-}T}$	S_t	c
1	-1.80	-2.21	1.55	1.77	1.95	0.84	0.98	0.54	0.60	4.59	-1.63	-0.80	-4.19	-4.39	-2.13	-3.07	-1.52	-1.92	-2.12	-3.98	1.58	-1.82	2.02	-0.54	0.36	1.91
2	-1.02	-1.77	2.02	1.82	2.57	2.48	1.44	0.56	0.60	8.47	-2.06	-1.32	-3.95	-3.11	-1.97	-2.10	-1.30	-1.24	-6.91	-2.12	0.78	-0.22	0.95	-0.44	0.23	4.82
3	-2.25	-2.45	1.95	0.05	2.23	1.11	1.13	0.53	0.57	5.46	-2.14	-1.23	-4.64	-3.63	-1.77	-2.88	1.06	-1.02	-8.28	-8.41	1.19	-6.51	2.26	-1.29	0.14	1.43
4	-0.88	-1.36	1.77	1.35	2.63	1.07	1.67	0.44	0.55	7.72	-1.14	-1.01	-4.08	-4.19	-2.22	-2.46	-2.05	-1.85	1.77	-1.13	1.34	-0.39	2.74	0.24	0.18	2.76
5	-1.71	-1.84	0.58	0.32	0.66	0.41	1.25	0.53	0.70	7.83	-0.99	0.78	-3.49	-3.15	-1.84	-2.40	-2.23	-2.41	1.69	1.82	1.34	0.62	0.70	0.02	0.50	3.80
6	-2.49	-2.61	3.01	0.70	3.27	0.99	1.42	0.69	0.66	4.46	-2.74	-0.67	-1.81	-3.67	-4.37	-3.05	-2.67	-2.55	1.84	-3.15	0.25	-2.50	2.07	-0.91	0.20	2.53
7	-2.10	-2.39	2.47	-0.29	2.35	-0.07	1.48	0.36	0.50	4.72	-2.60	-0.73	-4.75	-3.88	-1.96	-2.88	-0.71	-2.38	-4.44	-4.90	1.48	-2.18	2.07	-0.92	0.22	2.01
8	-1.39	-2.27	1.01	1.35	1.43	1.53	1.30	0.52	0.63	5.62	-1.85	0.06	-4.48	-3.82	-2.21	-3.17	-2.77	-2.93	-4.54	-2.21	0.65	-1.46	1.25	0.04	0.58	2.79
9	-1.61	-1.76	-0.19	-1.02	-0.31	-1.04	1.64	0.36	0.60	8.96	-2.09	-0.57	-5.21	-4.44	-2.00	-2.25	-2.33	-1.43	-4.65	0.99	1.42	1.66	3.52	2.61	0.59	2.77
10	-1.53	-1.98	0.74	0.98	1.89	1.89	0.80	0.28	0.64	4.72	-1.95	-0.93	-4.13	-3.97	-2.04	-3.62	-1.45	-2.29	-4.23	-8.48	1.07	-6.90	1.73	-1.14	0.40	2.44
11	-0.74	-0.67	1.25	0.67	1.42	1.03	1.42	0.42	0.62	7.21	-2.09	-0.62	-2.46	-3.78	-1.78	-2.87	-1.45	-2.34	5.18	7.96	3.98	5.93	3.52	3.93	0.58	2.58
12	-1.84	-1.59	2.21	0.23	4.01	1.46	1.14	0.41	0.61	6.82	-1.78	-0.44	-3.73	-3.34	-1.82	-2.36	-1.79	-2.06	4.74	-0.18	1.97	-0.44	2.88	1.02	0.47	4.39
13	-0.97	-2.10	0.13	-0.62	0.90	-0.06	1.53	0.40	0.54	7.87	-2.02	-0.09	-4.03	-3.74	-2.11	-2.52	-1.79	-2.42	-6.96	-2.56	-0.93	-1.79	-0.82	-3.45	0.60	3.39
14	-0.87	-1.19	2.34	1.77	2.28	0.83	0.92	0.43	0.69	7.92	-1.42	0.72	-4.25	-3.91	-1.24	-2.60	-0.31	-1.73	1.20	-1.71	1.00	-1.44	1.47	0.16	0.50	2.55
15	-1.55	-2.03	1.56	0.25	2.29	0.88	1.12	0.36	0.61	4.53	-2.05	-0.21	-4.17	-3.84	-2.58	-3.06	-2.76	-2.30	-3.27	-6.92	0.62	-4.43	1.48	-1.43	0.56	2.60
16	-1.79	-2.42	2.17	0.19	1.54	0.06	1.26	0.46	0.64	9.26	-2.78	-0.64	-3.73	-3.36	-1.93	-1.96	-0.60	-1.84	1.35	-2.84	1.92	-0.77	2.29	-0.10	0.26	3.15

17	-2.03	-1.97	-0.28	0.02	0.99	1.19	1.38	0.38	0.62	7.32	-1.87	-1.09	-4.15	-2.80	-1.01	-1.30	-0.42	-0.54	-9.30	-4.59	0.98	-1.25	1.35	-0.67	0.21	2.88
18	-2.24	-2.29	1.31	-0.22	2.34	0.32	1.34	0.50	0.61	8.18	-2.00	-0.94	-1.54	-2.29	-0.02	-1.28	1.29	-0.83	5.92	6.73	3.65	3.35	3.19	2.63	0.18	2.85
19	-1.42	-2.31	3.64	0.60	3.73	1.68	0.80	0.38	0.52	5.20	-1.73	-0.55	-3.86	-4.06	-1.28	-3.27	-0.30	-2.22	-1.48	-4.58	1.31	-4.83	1.51	-3.10	0.45	2.81
20	-1.46	-2.47	1.16	0.30	1.58	0.17	0.90	0.59	0.71	5.76	-2.03	-1.14	-3.94	-3.77	-2.83	-3.15	-2.96	-2.88	-3.86	-6.07	-0.92	-4.99	-0.14	-3.61	0.49	2.96
21	0.59	-0.53	1.40	1.27	1.81	0.74	1.21	0.60	0.51	7.43	-0.76	-1.06	-3.63	-3.55	-2.84	-1.71	-2.03	0.03	-4.88	-5.71	-4.07	-3.41	-3.75	-2.25	0.58	3.87
22	-2.22	-2.84	2.30	-1.39	3.10	0.20	1.66	0.42	0.58	6.24	-1.50	-0.19	-4.35	-3.34	-1.65	-2.20	-1.29	-2.08	-1.93	-2.60	2.30	-0.01	2.41	0.36	0.22	3.23
23	0.29	-1.63	1.31	1.83	1.30	1.21	1.31	0.74	0.60	5.60	-0.86	-0.58	-3.14	-3.26	-3.79	-2.52	-2.38	-2.95	-3.69	-5.65	-2.04	-2.78	-0.50	-2.68	0.56	3.34
24	-1.56	-2.51	3.09	0.44	2.19	0.65	1.24	0.47	0.57	6.10	-3.01	-1.67	-2.40	-2.50	-1.57	-2.05	-0.01	-1.06	-1.91	-6.28	-0.22	-4.77	1.36	-2.16	0.21	2.59

Note. P = Participant number

Supplementary Table 4. Model 3 median parameter estimates of the posterior distributions across participants.

p	$V_{HF\text{-}L}$	$V_{LF\text{-}L}$	$V_{HF\text{-}D\text{-}T}$	$V_{LF\text{-}D\text{-}T}$	$V_{HF\text{-}S\text{-}T}$	$V_{LF\text{-}S\text{-}T}$	a	z	T_{er},	σ	η_L	η_T	$\beta_{LPC\text{-}L}$,	$\beta_{P1\text{-}L}$	$\beta_{LPC\text{-}T}$	$\beta_{P1\text{-}T}$	$\beta_{0\text{-}LPC\text{-}L}$	$\beta_{0\text{-}P1\text{-}L}$	$\beta_{0\text{-}LPC\text{-}T}$	$\beta_{0\text{-}P1\text{-}T}$	S_t	c_{LPC}	c_{P1}
1	-1.97	-2.20	1.43	1.63	1.67	1.00	1.03	0.55	0.56	2.07	-0.75	-0.18	-2.99	-3.32	-2.44	-3.54	1.16	0.12	2.10	0.21	0.26	4.58	3.94
2	-0.97	-1.59	2.10	1.79	2.75	2.47	1.36	0.54	0.59	3.15	-1.90	-1.15	-1.11	-1.54	-2.55	-3.70	0.67	0.39	2.62	-1.54	0.22	7.79	6.16
3	-2.38	-2.28	2.14	-0.11	2.32	1.23	1.01	0.53	0.58	1.35	-2.59	-1.55	-2.90	-2.71	-1.68	-3.48	-1.07	-5.34	2.60	1.01	0.14	5.92	3.46
4	-0.97	-1.35	1.55	1.28	2.43	0.86	1.55	0.45	0.55	2.21	-0.94	-1.03	-1.71	-2.36	-2.36	-3.54	1.77	0.96	4.13	5.45	0.18	7.40	5.10
5	-1.56	-1.58	0.43	0.32	0.85	0.39	1.25	0.51	0.66	4.16	-0.67	0.46	-1.36	-1.95	-2.73	-3.51	0.85	0.33	3.70	0.64	0.37	7.12	7.00
6	-2.63	-2.59	2.95	0.47	2.82	1.01	1.27	0.69	0.66	2.41	-2.78	-0.88	-3.16	-2.39	-0.99	-2.22	1.80	-1.08	0.48	-6.76	0.20	4.43	4.85
7	-2.31	-2.41	2.08	-0.62	1.83	-0.13	1.29	0.39	0.50	1.56	-2.58	-1.27	-2.08	-3.12	-1.69	-2.44	1.19	-1.32	-0.46	-6.35	0.21	5.08	6.46
8	-1.56	-2.10	1.12	0.73	0.78	0.97	1.38	0.54	0.56	2.14	-1.09	-0.23	-2.58	-3.41	-2.27	-3.78	-0.40	0.43	1.08	-3.16	0.39	6.01	3.67
9	-1.41	-1.55	0.06	-0.87	-0.49	-1.11	1.62	0.38	0.54	2.87	-2.36	-0.76	-0.92	-1.87	-1.36	-3.93	1.61	3.19	1.52	0.10	0.45	7.87	5.10
10	-1.99	-1.71	-0.15	-0.03	1.28	1.33	0.87	0.35	0.59	2.75	-1.90	-0.24	-2.68	-3.62	-2.57	-3.63	0.65	-3.88	1.49	-2.87	0.31	4.61	4.67
11	-0.73	-0.85	0.66	0.50	0.93	0.57	1.42	0.46	0.56	1.72	-1.94	-0.97	-0.91	-1.87	-2.46	-1.91	3.44	4.77	-2.86	-3.30	0.40	7.21	6.35
12	-1.80	-1.64	2.36	0.11	3.40	1.05	1.17	0.43	0.56	3.76	-1.31	-0.12	-2.37	-1.97	-1.23	-2.72	3.44	1.36	2.26	-0.85	0.33	7.09	7.80
13	-1.05	-1.66	0.02	-0.47	0.45	-0.06	1.57	0.42	0.46	3.39	-2.20	-0.50	-0.87	-2.25	-1.15	-2.68	-0.52	-2.94	2.51	2.78	0.54	7.37	6.54
14	-0.94	-1.17	1.54	0.91	1.24	0.42	1.01	0.47	0.62	2.36	-1.52	-0.35	-1.40	-1.90	-1.78	-2.54	1.42	0.72	-2.17	-6.39	0.36	7.40	4.72
15	-1.57	-2.16	1.04	-0.31	1.64	0.11	1.21	0.44	0.54	2.02	-1.79	-0.14	-2.78	-2.68	-2.52	-3.58	0.31	-2.51	0.40	-6.40	0.35	4.71	5.00
16	-1.73	-2.15	2.56	0.10	1.50	0.06	1.12	0.45	0.64	2.56	-2.73	-0.16	-0.43	-2.31	-1.09	-2.79	1.97	-0.12	0.73	-5.98	0.26	7.91	4.29
17	-2.15	-1.81	-0.32	0.10	0.98	1.05	1.23	0.38	0.63	3.27	-1.79	-0.83	-2.20	-0.47	-2.40	-4.14	-1.49	-0.89	0.93	-3.24	0.24	7.09	5.58

18	-2.05	-2.29	1.81	-0.29	2.46	0.31	1.17	0.49	0.62	2.22	-2.17	-0.56	0.82	-0.86	-2.80	-3.79	3.69	4.02	2.82	-0.71	0.19	7.56	5.40
19	-1.60	-2.08	2.90	0.06	2.99	1.18	0.90	0.42	0.46	1.71	-1.45	-0.39	-2.89	-3.18	-1.30	-2.67	-0.92	-4.01	0.63	-4.59	0.32	5.91	6.14
20	-1.51	-2.12	0.67	0.02	0.98	0.22	0.95	0.61	0.66	2.70	-1.82	-1.39	-2.95	-2.84	-2.25	-2.92	-2.44	-4.90	3.36	2.95	0.36	5.76	4.59
21	0.58	-0.09	1.32	1.52	1.51	1.21	1.25	0.56	0.47	4.19	-0.96	-1.11	-1.69	-0.94	-2.10	-1.97	-3.68	-3.71	0.39	-0.53	0.57	7.09	5.82
22	-2.55	-2.81	2.74	-1.66	3.14	-0.13	1.60	0.43	0.58	2.39	-1.26	0.26	-1.97	-2.04	-2.39	-3.85	2.25	0.45	2.86	-0.14	0.23	6.45	4.22
23	0.17	-1.34	0.96	1.72	0.90	1.09	1.34	0.72	0.54	3.05	-0.74	-0.84	-2.92	-1.89	-3.70	-3.34	-2.40	-2.39	1.93	-0.24	0.44	5.43	4.82
24	-1.95	-2.27	3.20	0.33	2.81	1.10	1.18	0.47	0.57	2.89	-1.92	-0.54	-2.16	-1.81	-0.17	-3.29	-2.79	-4.80	2.59	0.92	0.20	6.20	5.23

Note. P = Participant number

Supplementary Table 5. Model 4 median parameter estimates of the posterior distributions across participants.

Participant Number	V_{HF-L}	V_{LF-L}	V_{HF-D-T}	V_{LF-D-T}	V_{HF-S-T}	V_{LF-S-T}	a	z	T_{er},	σ	η_L	η_T	β_L	β_T	β_{oL}	β_{oT}	S_t
1	-2.29	-2.51	1.14	1.55	1.63	1.01	1.12	0.58	0.53	5.10	-0.43	0.32	-2.15	-2.61	-0.36	-0.78	0.40
2	-0.94	-1.78	2.68	2.33	3.61	3.12	1.45	0.53	0.60	10.08	-1.06	-0.14	-1.83	-0.96	0.51	-1.12	0.24
3	-3.26	-2.85	2.87	-0.38	3.56	1.66	1.12	0.54	0.58	6.23	-0.74	0.40	-1.72	-1.83	-2.15	-1.39	0.17
4	-1.07	-1.76	1.84	1.38	3.21	1.21	1.64	0.43	0.56	8.71	-0.30	-0.14	-1.53	-1.52	0.26	-0.21	0.22
5	-1.55	-1.85	0.41	0.01	0.81	0.28	1.40	0.54	0.62	8.78	-0.74	0.61	-1.75	-2.17	-0.18	-1.13	0.50
6	-2.99	-2.81	3.49	0.53	3.84	1.05	1.38	0.70	0.66	5.18	-1.13	-0.13	-1.99	-2.63	-0.47	-0.82	0.21
7	-2.75	-2.84	2.81	-1.18	2.94	-0.25	1.57	0.37	0.49	5.40	-1.25	0.43	-1.91	-2.25	-0.84	-1.22	0.28
8	-1.51	-1.91	1.16	0.86	0.85	1.06	1.57	0.54	0.51	6.41	-1.34	0.13	-1.90	-2.06	-0.45	-1.34	0.51
9	-1.58	-1.94	-0.33	-1.17	-0.96	-1.53	1.82	0.44	0.50	10.22	-1.85	-0.24	-1.81	-1.18	0.50	-0.38	0.53
10	-1.98	-1.67	-0.58	0.00	1.56	1.65	0.92	0.35	0.57	5.45	-1.98	0.57	-2.20	-1.94	-1.41	-1.18	0.40
11	-0.64	-1.03	1.00	0.66	1.27	0.68	1.67	0.45	0.51	7.92	-1.33	-0.07	-0.61	-0.45	0.81	0.86	0.51
12	-1.39	-1.41	2.12	-0.06	3.37	1.10	1.27	0.40	0.52	8.14	-1.99	-0.64	-1.79	-1.06	0.41	0.04	0.48
13	-1.27	-2.17	-0.10	-0.88	0.22	-0.61	1.75	0.46	0.46	9.22	-1.27	0.48	-1.06	-1.67	-1.84	-1.84	0.52
14	-0.70	-1.10	2.16	1.26	2.02	0.91	1.12	0.42	0.60	9.02	-1.61	1.11	-1.87	-1.36	-0.03	-0.39	0.45
15	-1.51	-2.04	1.30	-0.49	2.12	-0.07	1.43	0.42	0.49	5.16	-1.57	0.50	-1.99	-2.64	-2.24	-1.04	0.49
16	-2.23	-2.51	3.14	-0.27	2.20	0.12	1.28	0.45	0.64	10.30	-1.22	1.48	-1.85	-1.97	-0.42	-0.57	0.28

17	-2.29	-1.83	-0.50	-0.23	0.98	1.17	1.29	0.39	0.62	8.22	-1.45	-0.38	-1.13	-1.96	-1.53	-1.70	0.23
18	-3.01	-3.42	2.76	-0.78	3.55	0.44	1.36	0.50	0.62	9.31	-0.48	0.78	0.11	0.28	1.17	0.70	0.24
19	-1.76	-1.95	2.99	-0.06	3.75	1.34	1.02	0.41	0.44	6.21	-1.02	0.27	-1.98	-1.93	-1.27	-1.35	0.38
20	-0.97	-1.63	1.13	0.58	1.61	0.63	1.11	0.52	0.58	6.35	-2.18	-0.54	-2.89	-1.83	-2.13	-4.75	0.51
21	1.11	0.02	1.64	2.60	2.23	2.11	1.43	0.54	0.46	8.71	0.84	0.31	-1.01	-1.33	-2.51	-1.95	0.56
22	-2.81	-3.03	3.37	-1.90	3.47	-0.23	1.69	0.42	0.58	6.92	-0.81	0.66	-1.96	-1.56	0.02	-0.77	0.24
23	0.54	-1.04	1.35	2.11	1.15	1.92	1.49	0.66	0.51	6.23	-0.20	-0.36	-1.93	-1.70	-1.20	-1.32	0.49
24	-2.19	-2.31	3.52	0.39	3.59	1.72	1.29	0.44	0.56	7.00	-1.07	0.41	-1.03	-1.33	-2.03	-1.88	0.24
